# A Novel RET D898Y Germline Mutation in a Patient with Pheochromocytoma

**DOI:** 10.1155/2018/8657914

**Published:** 2018-04-15

**Authors:** Jin Wook Yi, Hye In Kang, Su-jin Kim, Chan Yong Seong, Young Jun Chai, June Young Choi, Moon-Woo Seong, Kyu Eun Lee, Sung Sup Park

**Affiliations:** ^1^Department of Surgery, Seoul National University Hospital and College of Medicine, 101 Daehak-ro, Jongno-gu, Seoul 110-744, Republic of Korea; ^2^Cancer Research Institute, Seoul National University College of Medicine, 101 Daehak-ro, Jongno-gu, Seoul 110-744, Republic of Korea; ^3^Department of Surgery, Seoul National University Boramae Medical Center, 20 Boramae-ro 5-gil, Dongjak-gu, Seoul 156-70, Republic of Korea; ^4^Department of Surgery, Seoul National University Bundang Hospital, Seoul National University College of Medicine, Seongnam, Republic of Korea; ^5^Department of Laboratory Medicine, Seoul National University Hospital and College of Medicine, 101 Daehak-ro, Jongno-gu, Seoul 110-744, Republic of Korea

## Abstract

Pheochromocytoma and paraganglioma are tumors of neuroectoderm origin. Up to 40% of patients with these tumors have germline mutations in known susceptibility genes. We report a novel* RET* germline mutation (exon 15; c.2692G>T (D898Y)) in a pheochromocytoma patient, as well as in her two asymptomatic sons and older sister. A 49-year-old female came to our clinic presenting with a right adrenal gland mass detected during a healthcare examination. Her mother and two sisters had previously undergone thyroidectomy for papillary thyroid carcinomas. The levels of vanillylmandelic acid and other catecholamines were elevated in 24-hour urine, and an imaging study revealed a right adrenal mass. She underwent laparoscopic adrenalectomy and the final pathologic diagnosis was pheochromocytoma. Mutation screening detected a* RET* p.D898Y mutation, both in the patient and in the patient's two sons and older sister. This is the first description of a* RET* D898Y mutation in a pheochromocytoma patient and her family. The mutation should be categorized as a variant of unknown significance because no RET gene related disorders were detected in this family. Long term follow-up will be required to determine the clinical significance of the* RET* D898Y mutation.

## 1. Introduction

Pheochromocytomas (PCCs) and paragangliomas (PGLs) are chromaffin cell origin neuroendocrine tumors. PCCs arise from the adrenal medulla and produce catecholamines, whereas paragangliomas occur in the thoracoabdominal sympathetic or parasympathetic ganglia and may, or may not, be associated with catecholamine secretion [1]. World Health Organization tumor classification (2004) defined PCC as an intra-adrenal paraganglioma, due to evidence for shared genetic predisposition to these tumors [2]. With the advancement of molecular pathogenesis, germline mutations in pheochromocytoma and/or paraganglioma (PPGL) susceptibility genes, such as* NF1*,* RET*,* VHL*,* SDHD*,* SDHC*,* SDHB*,* SDHAF2*,* SDHA*,* TMEM127*,* MAX*,* EPAS1*, and* FH*, have been identified during the past 15 years. The total incidence of germline mutations in both familial and sporadic PPGL is up to 40% [3–6].

Among the many PPGL susceptibility genes,* RET* is a well-known protooncogene, germline mutations of which cause multiple endocrine neoplasia type 2 (MEN2), which is characterized by medullary thyroid carcinoma (MTC), PCC, and hyperparathyroidism [7, 8]. Almost 100% of MEN2 patients will develop MTC, and there is a 50% risk of PCC penetrance in their lifetime [9–12]. To date (2018), over 100 genetic alterations in* RET* have been reported and registered in the “*RET* protooncogene database” [13], and the penetrance of* RET* mutation-related diseases varies depending on the site of the* RET* mutation [10]. In patients with some* RET* gene mutation types, the occurrence of PCC as an initial clinical manifestation is more frequent than the occurrence of MTC [14–18]. For this reason, mutation screening of* RET* in PPGL patients and their families is important for the establishment of appropriate treatment management plans [10, 11].

In the present study, a novel germline* RET* gene mutation (c.2692G>T, p.Asp898Tyr) of unknown significance was identified in a patient with PCC and her family members at the Seoul National University Hospital, South Korea. We evaluated the genomic features and familial characteristics of the mutation using a computational biology tool. This study was approved by the Institutional Review Board of Seoul National University Hospital (IRB number: H-1677-006-772).

## 2. Methods

### 2.1. Case Presentation

**Figure 1:**
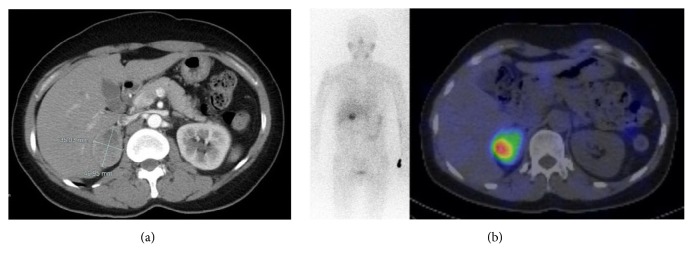
(a) Abdominal computed tomography showing a heterogeneous enhanced mass in the right adrenal gland. (b) Image generated by scintigraphy and single-photon emission computed tomography showing the right adrenal mass and demonstrating increased uptake of metaiodobenzylguanidine.

In November 2012, a 49-year-old Korean woman was referred to the Department of Endocrine Surgery due to a right adrenal mass, identified by abdominal sonography during a routine healthcare checkup. She had been suffering from headache, tachycardia, palpitations, and cold sweats for 5 years but had not previously undergone adrenal disease related diagnostic tests. Her mother and two sisters had a history of thyroidectomy as treatment for papillary thyroid cancer. Twenty-four-hour urine collection revealed elevated vanillyl mandelic acid (VMA; 11.7 mg/day, reference: 2–7 mg/day), metanephrine (1625.6 *μ*g/day, reference: 52–341 *μ*g/day), normetanephrine (5459.5 *μ*g/day, reference: 88–444 *μ*g/day), epinephrine (256.4 *μ*g/day, reference: 0.20 *μ*g/day), and norepinephrine (153.2 *μ*g/day, reference: 15–80 *μ*g/day). An abdominal computed tomography (CT) scan revealed a heterogeneous right adrenal mass of approximately 4.7 × 3.5 cm (Figure 1(a)). Metaiodobenzylguanidine scintigraphy and single-photon emission CT showed increased uptake in the right adrenal gland (Figure 1(b)). The patient underwent right adrenalectomy using the posterior retroperitoneoscopic approach. The final pathologic diagnosis was a PCC with a size of 6.0 × 4.0 × 3.0 cm and a high risk of malignancy with a “PCC of the adrenal gland scaled score” of 13.

During the 65-month follow-up after adrenalectomy, the levels of urine VMA, catecholamine, serum calcitonin, calcium, carcinoembryonic antigen, and parathyroid hormone remained within normal ranges. There was no evidence of malignant thyroid nodules on neck ultrasound examination. Genetic counseling was performed by a specialist nurse. Due to the family history of thyroid cancer, we suggested germline mutation screening of the* RET* gene to the patient and her family members. The index patient and her two sons, two younger brothers, one younger sister, and mother agreed to the germline mutation test and provided informed consent. The informed consent statement contained a statement concerning the sharing of results as follows: “The research, which was conducted for your information or human tissue samples, can be shared with other researchers through congress. We can publish the results so that other interested people can learn from this study.”

### 2.2. Mutation Analysis

Genomic DNA was extracted from peripheral blood samples from the index patient and her family members. For the index patient, mutation testing included* RET* gene exons 8, 10, 11, 13, 14, 15, and 16 and their flanking regions using PCR and direct sequencing of DNA. For the family members, direct sequencing was performed only for* RET* exon 15. The GenBank reference sequence used to analyze data generated by direct sequencing was NM_020975.4. The Ensembl VEP (variant effect predictor, http://www.ensembl.org/info/docs/tools/vep/index.html) program was used to predict the biological effect of single nucleotide variants. To predict the impact of amino acid changes on protein structure and function, we used the SIFT (http://sift.jcvi.org/) and PolyPhen (http://genetics.bwh.harvard.edu/pph2/bgi.shtml) prediction tools. The ExAC database (http://exac.broadinstitute.org/) was used to identify the frequency of novel single nucleotide variants in the general population. To evaluate the clinical significance of previously reported* RET* gene variants, we exploited the dbSNP (https://www.ncbi.nlm.nih.gov/SNP/) and ClinVar (https://www.ncbi.nlm.nih.gov/clinvar/) databases.

## 3. Results

**Figure 2:**
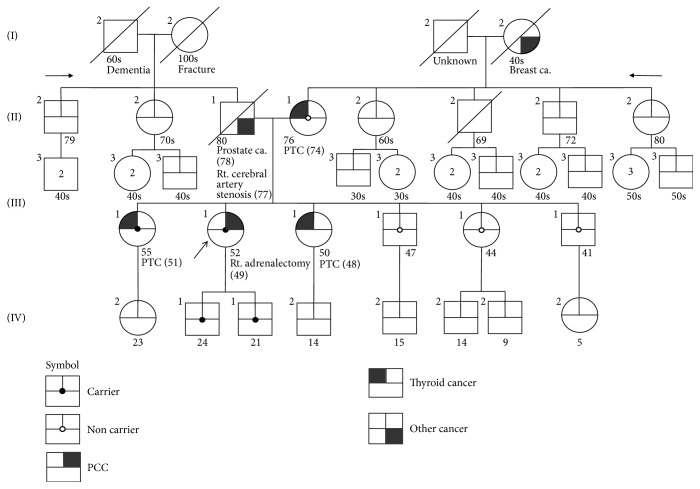
Pedigree of the index patient (arrow) and her family. The* RET* D898Y germline mutation was detected in the index patient, her two sons, and her older sister.

**Figure 3:**
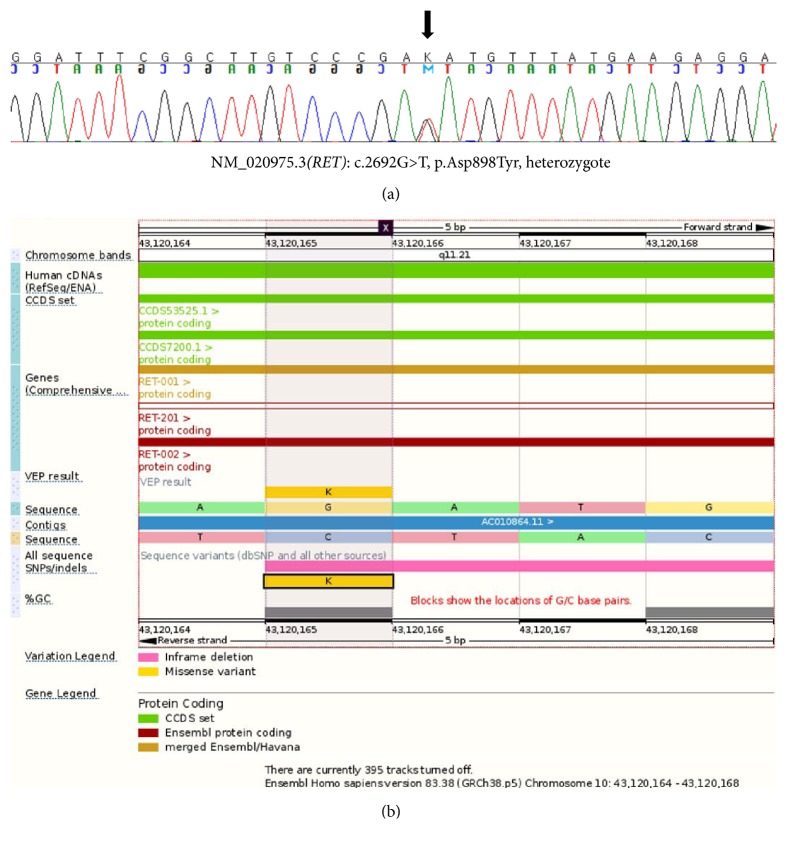
(a) Sanger sequencing revealed a* RET* mutation (c.2692G>T, p.Asp898Tyr) (chromosome 10:43120165). (b) Detailed information on the RET c.2692G>T, p.Asp898Tyr mutation generated by variant effect predictor (http://www.ensembl.org/info/docs/tools/vep/index.html).

A germline mutation in the* RET* gene was identified in the index patient, her two sons, and her older sister. It was not identified in the mother or two younger brothers and one sister of the patient (Figure 2). Sanger sequencing revealed that the* RET* gene mutation was a c.2692G>T substitution (chromosome 10:43120165, reference sequence, GRCh38.p5), (Figure 3(a)). VEP analysis indicated that the mutation alters a GAT codon to TAT, resulting in a change in codon 898 (p.D898Y, Figure 3(b)). The score of the change according to the SIFT tool was 0, indicating that it is predicted to be “deleterious.” The PolyPhen score was 1, indicating that it is predicted as “probably damaging.” The frequency of the mutant allele in the population, estimated using the ExAC database, was 1.648*e* − 05, indicating that this is an extremely rare allele. Her sister and two sons, who also carried the mutation, did not develop any tumors during the 65-month surveillance period, although her sister had a history of thyroid cancer (Figure 2).

## 4. Discussion

The* RET* protooncogene is a member of the cadherin superfamily and encodes the RET transmembrane tyrosine kinase protein.* RET* maps to chromosome 10q11.2 and consists of 55,000 base pairs including 21 exons [19]. Mutations of* RET* are associated with MEN types 2A and 2B and familial MTC [7]. Hundreds of* RET* mutations have been identified, and the penetrance and aggressiveness of MEN related tumors vary according to the specific mutations [20]. For example, mutation of* RET* codon 634 is the most frequently identified mutation in western countries and is associated with increased aggressiveness and younger onset of MTC compared with other* RET* mutations. Prophylactic thyroidectomy is recommended for children under 5 years old carrying the* RET* 634 mutation [21, 22]. The American Thyroid Association guidelines (2015) for managing MTC list the relationships between important* RET* gene mutations and aggressiveness of MTC and incidence of PCC and other related tumors, in order to provide clinical guidance on the management of MEN patients [10].

Here, we describe the first* RET* D898Y germline mutation identified in a PCC patient and her two first-degree relatives. The* RET* D898Y mutation was previously reported once in dbSNP (rs587780810) and once in the ClinVar database (RCV000123314.1) as a variant of uncertain significance. There is no other clinical reports describing the* RET* D898Y mutation in the literature. Judging from the pedigree and sequencing results (Figures 2 and 3), this mutation has an autosomal dominant inheritance pattern with heterozygosity, although the index patient and family members did not show diagnostic features characteristic of MEN or MEN related diseases. PCC was also found in the index patient. The genotype-phenotype correlation and penetrance of MEN related tumors or PCC have not been proven. We suggest that this mutation be classified as a novel germ line mutation of unknown significance. Long term surveillance of this patient and her relatives will be needed to reveal genotype-phenotype correlations of this mutation. A follow-up plan for the index patient and her family members carrying* RET* D898Y should consist of yearly checkups, including assaying for parathyroid hormone, carcinoembryonic antigen, calcitonin, 24-hour urine catecholamine, and VMA levels, as well as screening using thyroid ultrasound to detect PCC, MTC, or hyperparathyroidism.

The prevalence of germline mutations in PPGL is reported as approximately 40%, and recent genomic studies report that the incidence of overall germline plus somatic mutation in PPGL is as high as 60% [3–6]. Using next generation sequencing and transcriptome analysis, researchers have identified many PPGL susceptibility genes, which are classified into two clusters: cluster 1 (the angiogenic cluster) consists of* EGLN1*,* PHD2*,* VHL*,* SDHX*,* IDH*,* HIF2A*,* EPAS1*,* FH*, and* MDH2*, while cluster 2 (the kinase signaling cluster) includes* KIF1B*,* RET*,* NF1*,* TMEM127*,* MAX*, and* MEN1* [4, 23]. For these reasons, genetic counseling and mutation screening tests of all PPGL patients are essential for proper management of multiple endocrine tumors and can modulate surgical treatment plans and early stage detection of related tumors and allow the use of medication targeted for specific mutations [24, 25].

In the Seoul National University Hospital, PPGL patients undergo genetic counseling with a specialist nurse. The nurse takes their family histories and draws pedigrees. Germline mutation tests are performed on index patients and family members with their informed consent. At present (2018), we operate a mutation panel test for specific PPGL patients as part of a nationalized rare disease diagnosis support program. Patients who have one of the following criteria can enroll in this program: age under 50 years, family history of PPGL, and multiple or bilateral tumors, recurrent PPGL, or malignant tumors diagnosed by pathology. Targeted sequencing of ten PPGL susceptibility genes,* MAX*,* NF1*,* RET*,* SDHA*,* SDHAF2*,* SDHB*,* SDHC*,* SDHD*,* TMEM127*, and* VHL*, is performed with the support of funds from a national budget (Table 1).

Recent advances in next generation sequencing technology have produced a cost-effective method to discover novel mutations responsible for hereditary disease. Whole exome sequencing or targeted next generation sequencing has a faster sequencing time and lower cost than conventional Sanger sequencing when performing analysis of multiple candidate genes, such as that required for PPGL [26–28]. Another analytic platform, RNA sequencing, can not only detect novel sequence alterations (single nucleotide variants, indels, and chromosomal rearrangements), but can also quantify gene expression through analysis of read alignment counts, allowing more comprehensive analysis [26]. However, analysis of whole exome sequence or targeted next generation sequencing data requires more complex analytic pipelines and bioinformatics expertise than direct sequencing analysis.

## 5. Conclusions

We identified a D898Y mutation in the* RET* gene with autosomal dominant inheritance in a PCC patient and her first-degree relatives. Monitoring the development of PPGL and MTC in the patient and carrier family members is ongoing; hence the penetrance of tumors is unknown. Mutational screening using targeted sequencing is important for proper management of PPGL patients and their families. Next generation sequencing is likely to be more helpful for detection of novel mutations than classic direct sequencing.

## Figures and Tables

**Table 1:** Paraganglioma/pheochromocytoma genetic mutational panel screened in the rare disease control program in Korea.

Gene	Reference sequence
*MAX*	NG_029830.1, NM_002382.4
*NF1*	NG_009018.1, NM_000267.3
*RET*	NG_007489.1, NM_020975.4
*SDHA*	NG_012339.1, NM_004168.2
*SDHAF2*	NG_023393.1, NM_017841.2
*SDHB*	NG_012340.1, NM_003000.2
*SDHC*	NG_012767.1, NM_003001.3
*SDHD*	NG_012337.2, NM_003002.2
*TMEM127*	NG_027695.1, NM_017849.3
*VHL*	NG_008212.3, NM_000551.3
